# Socioeconomic Status and Overweight: A Population-Based Cross-Sectional Study of Japanese Children and Adolescents

**DOI:** 10.2188/jea.JE20140108

**Published:** 2015-07-05

**Authors:** Yuko Kachi, Toshiaki Otsuka, Tomoyuki Kawada

**Affiliations:** Department of Hygiene and Public Health, Nippon Medical School, Tokyo, Japan

**Keywords:** socioeconomic status, overweight, children, adolescent, Japan

## Abstract

**Background:**

Socioeconomic status (SES) as a determinant of obesity has received scant attention in Japan. This study examined the association between SES and overweight among Japanese children and adolescents.

**Methods:**

Cross-sectional analyses of a representative sample of Japanese children (6–11 years: *n* = 397) and adolescents (12–18 years: *n* = 397) were performed, with measured heights and weights from the 2010 National Health and Nutrition Examination Survey and the 2010 Comprehensive Survey of Living Conditions. Overweight, including obesity, was defined by International Obesity Task Force cut-offs. SES indicators included household income, equivalent household expenditure, parental educational attainment, and parental occupational class.

**Results:**

Overweight prevalence was 12.3% in children and 9.1% in adolescents. Adolescents living in middle-income households were more likely to be overweight than those living in high-income households (OR 2.26, 95% CI, 1.01–5.67) after adjustment for age, sex, and parental weight status. Similarly, adolescents living in households with low expenditure levels were more likely to be overweight than those living in households with high expenditure levels (OR 3.40, 95% CI, 1.20–9.60). In contrast, no significant association was observed among children.

**Conclusions:**

Our results indicated that low household economic status was associated with being overweight, independent of parental weight status, among Japanese adolescents.

## INTRODUCTION

Childhood and adolescent obesity presents a major public health challenge^1^ because it not only leads to medical and psychological complications, but also often leads to adult obesity and its associated morbidity and mortality.^2^^–^^4^ In Japan, the prevalence of overweight and obesity among children and adolescents greatly increased during the late 1970s to early 2000s and has remained high since.^5^ According to the Japanese-specific definition of overweight, 4%–11% of children and 9%–11% of adolescents were considered overweight in 2010.^5^ This prevalence cannot be compared directly with that in other countries due to differences in the definition of overweight and obesity. However, the secular trend is similar to that observed in other developed countries, such as the United States and the United Kingdom, where the prevalence remains stable at high levels.^6^^,^^7^ This implies that current efforts to reduce childhood and adolescent obesity rates have reached a plateau. Thus, there is an urgent need to develop more effective strategies to prevent childhood and adolescent obesity.

Many studies in developed countries have shown that low socioeconomic status (SES) is associated with obesity among children and adolescents.^8^^–^^11^ Among SES indicators, such as income, occupational class, and educational attainment, low maternal education has been most consistently associated with childhood and adolescent obesity.^9^ In Japan, SES as a determinant of obesity has received scant attention among public health officials, as Japan has long been considered an egalitarian society.^12^ However, the situation may be changing. Income inequality in Japan has risen steadily since the mid-1980s.^13^ In the face of widening socioeconomic disparities, researchers have begun to examine socioeconomic disparities in health, mostly among adults, since the late 1990s.^14^

Only a few Japanese studies have examined socioeconomic inequalities in obesity risk among children and adolescents, and findings have not been conclusive. An ecological study using prefecture-level data reported that the proportion of people completing up to college or university education was inversely associated with the prevalence of childhood obesity.^15^ By contrast, other studies using individual-level data reported that maternal educational attainment was not significantly associated with overweight^16^ or developmental body mass index (BMI) trajectories^17^ among children. These latter studies were not designed to determine the association between SES and adiposity and only included maternal educational attainment as a SES indicator.

The objective of this study was to examine the association between SES and objectively measured overweight among a nationally representative sample of Japanese children and adolescents. We used a range of SES indicators, including household income, household expenditure, parental educational attainment, and parental occupational class.

## METHODS

### Data sources

We used data from 2 nationally representative surveys conducted by the Ministry of Health, Labour and Welfare: the 2010 Comprehensive Survey of Living Conditions (CSLC)^18^ and the 2010 National Health and Nutritional Survey (NHNS).^19^ We obtained permission from the Ministry of Health, Labour and Welfare to use data from the two surveys.

Data on household expenditure and parental educational attainment were obtained from the 2010 CSLC. The CSLC has been collecting detailed information on household demographics annually since 1986 but only began collecting data on educational attainment in the 2010 survey. Eligible respondents included all members of households within census tracts that were randomly selected from prefectures and designated cities with a population over 500 000 people. A self-administered questionnaire was distributed to respondents in advance and later collected by trained investigators during home visits. The 2010 CSLC was conducted in 229 785 households across 5510 census tracts (response rate, 79%).

Data on objectively measured height and weight for children, adolescents, and their parents; household income; and parental occupational class were obtained from the NHNS. The NHNS has been collecting data on the health and nutritional status of the Japanese population annually since 1948. Eligible respondents included all household members aged 1 year or older within 300 census tracts, which were randomly selected from the above census tracts of the CLSC. The NHNS comprised a physical examination, including height and weight measurement by health care professionals, and self-administered questionnaire surveys on diet, lifestyle, and demographics (eg, household income and parental occupational class). The 2010 NHNS was conducted in 3684 households (response rate, 68%).

Referring to previous investigations,^20^ we linked data from the NHNS and CSLC using prefecture, area, household number, the number of household members, sex, and age, because both surveys share sampling units (Figure). Although the number of household members recruited in the NHNS was not released by the government, 9265 participants aged 6 years or older had physical examination records. We restricted our analyses to the 598 children who were aged 6 to 11 years and the 597 adolescents who were aged 12 to 18 years. Of these, 501 children and 547 adolescents could be linked to CSLC data. We then excluded 104 children and 150 adolescents for whom there were no height and weight data. A total of 397 children and 397 adolescents were ultimately included in this study. Ethical approval was not required, as the research involved retrospective analysis of a national surveillance dataset that was free of personally identifiable information.

**Figure.**
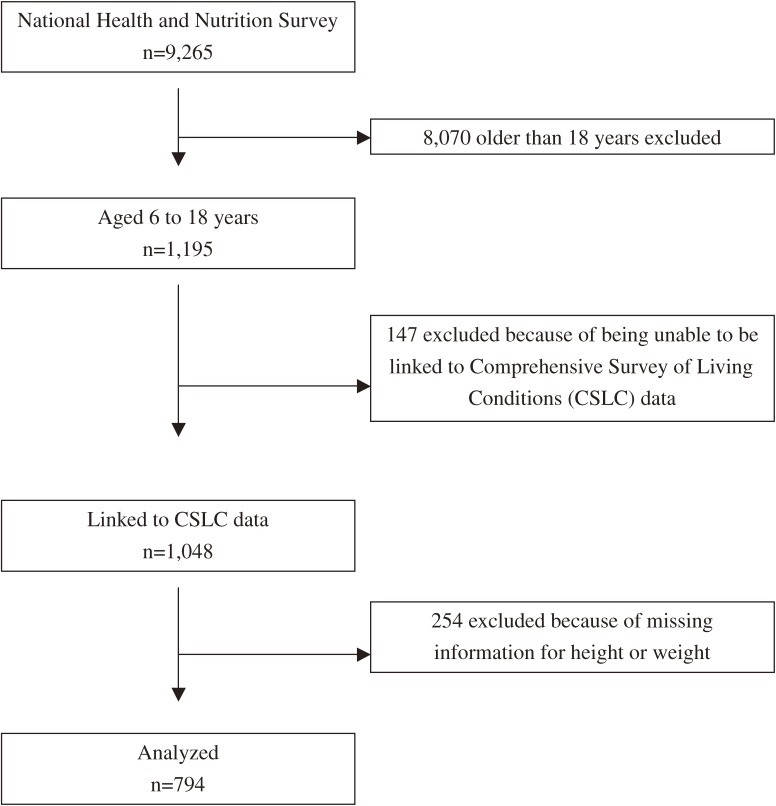
Flow diagram of included and excluded participants.

### Overweight prevalence

Height and weight were measured to the nearest 0.1 cm and 0.1 kg, respectively, in light clothing without shoes. BMI was calculated as weight in kilograms divided by the square of height in meters. Overweight was defined according to the International Obesity Task Force (IOTF) age- and sex-specific BMI cutoffs.^21^ IOTF criteria for overweight and obesity are based on identifying the childhood BMI thresholds that correspond to adult BMI thresholds of 25 and 30 kg/m^2^, respectively. We combined overweight and obese groups because of the small number of children and adolescents in the obese category.

### Socioeconomic status

SES indicators, such as household income, equivalent household expenditure, parental educational attainment, and parental occupational class were assessed via parent reports on the self-administered questionnaires. Taking our cue from previous research,^20^^,^^22^ we used household income and household expenditure as surrogate indicators of household economy. Household income in the past year was assessed using four categories: (1) less than 2 million yen (coded as low), (2) 2 to less than 6 million yen (middle), (3) 6 or more million yen (high), and (4) “Don’t know”. Equivalent household expenditure was calculated by dividing household expenditure per month by the square root of the household size.^23^ Equivalent monthly household expenditure was divided into tertiles, separately for children and adolescents, as follows: (1) low (20 000 to 90 000 yen for children and 20 000 to 100 000 yen for adolescents), (2) middle (94 000 to 140 000 yen and 103 000 to 150 000 yen, respectively), and (3) high (141 000 to 1 342 000 yen and 150 000 to 1 342 000 yen, respectively). Parental educational attainment was categorized into three groups: (1) less than high school (primary or junior high school), (2) completion of high school, and (3) greater than high school (technical college, two-year college, university, or graduate school). Parental occupational class was categorized into four groups according to the categories used in previous studies^24^: (1) professional or manager; (2) sales, service, or clerical; (3) security, transportation, or labor; and (4) others.

### Confounders

Confounders were selected on the basis of previous studies on this topic^9^ and included age (continuous), gender, and maternal weight status. Prior research has shown that parental overweight is a strong risk factor for childhood and adolescent overweight, which is the result of both genetic and environmental components.^25^ We selected maternal weight status rather than paternal weight status as a confounder because maternal BMI was more strongly correlated with a child’s BMI. Maternal BMI was calculated from measured height and weight. Maternal weight status was then classified according to BMI categories as underweight (<18.5 kg/m^2^), normal weight (18.5–24.9 kg/m^2^), and obesity (≥25.0 kg/m^2^), using the criteria of the Japan Society for the Study of Obesity.^26^

### Statistical analysis

We performed all analyses separately by age groups. Males and females were pooled because there was no strong evidence for gender-specific associations (for all interactions between gender and SES, *P* > 0.05).^9^ We applied a multilevel logistic regression analysis to examine the associations between SES and the risk of being overweight because participants were nested within households. This method takes into account the non-independence of observations within groups (ie, participants within households). The models controlled for age, gender, and maternal weight status. Variables with missing data were dummy coded using the missing-indicator method in the models. A sensitivity analysis using model-wise deletion was performed to compare the results obtained by different methods for handling missing data. All statistical tests were two-sided with a 5% significance level. All analyses were conducted using SAS Version 9.3 for Windows (SAS, Inc., Cary, NC, USA).

## RESULTS

The distribution of demographic and socioeconomic characteristics was similar between children and adolescents (Table 1). Approximately 15% of maternal BMIs fell into the obese category. Approximately 6% of participants lived in a household with an annual income of less than 2 million yen, and around 5% had a mother or father with less than a high school education. The majority of mothers were engaged in sales, service, or clerical occupations, while fathers were less likely to be engaged in such occupations.

**Table 1.** Participant characteristics by age group

	Aged 6 to 11 years	Aged 12 to 18 years
	(*n* = 397)	(*n* = 397)
	Mean ± SD or *n* (%)	Mean ± SD or *n* (%)
Age	8.7 ± 1.7	15.0 ± 1.9
Gender		
Male	210 (52.9)	203 (51.1)
Female	187 (47.1)	194 (48.9)
Height, cm	131.6 ± 10.9	160.7 ± 8.9
Weight, kg	29.0 ± 7.7	51.7 ± 10.7
Maternal weight status		
Underweight	42 (10.6)	55 (13.9)
Normal weight	269 (67.8)	243 (61.2)
Obese	58 (14.6)	60 (15.1)
Missing	28 (7.1)	39 (9.8)
Household income		
High	109 (27.5)	142 (35.8)
Middle	205 (51.6)	163 (41.1)
Low	23 (5.8)	23 (5.8)
“Don’t know”/Missing	60 (15.1)	69 (17.4)
Household expenditure		
High	125 (31.5)	124 (31.2)
Middle	129 (32.5)	129 (32.5)
Low	128 (32.2)	126 (31.7)
Missing	15 (3.8)	18 (4.5)
Maternal education		
>High school	207 (52.1)	186 (46.9)
High school	136 (34.3)	161 (40.6)
<High school	19 (4.8)	11 (2.8)
Missing	35 (8.8)	39 (9.8)
Paternal education		
>High school	176 (44.3)	154 (38.8)
High school	152 (38.3)	131 (33.0)
<High school	22 (5.5)	24 (6.1)
Missing	47 (11.8)	88 (22.2)
Maternal occupation		
Professional/manager	60 (15.1)	72 (18.1)
Sales/service/clerical	120 (30.2)	164 (41.3)
Security/transportation/labor	17 (4.3)	30 (7.6)
Others	167 (42.1)	91 (22.9)
Missing	33 (8.3)	40 (10.1)
Paternal occupation		
Professional/manager	120 (30.2)	111 (28.0)
Sales/service/clerical	82 (20.7)	67 (16.9)
Security/transportation/labor	111 (28.0)	92 (23.2)
Others	14 (3.5)	28 (7.1)
Missing	70 (17.6)	99 (24.9)

A total of 49 children (12.3%) and 36 adolescents (9.1%) were overweight. Table 2 shows the odds ratios (ORs) and 95% confidence intervals (CIs) of being overweight for children, as computed using multilevel logistic regression analysis. Children with an obese mother were more likely to be overweight (OR 2.72; 95% CI, 1.30–5.71) compared with their peers whose mothers were normal weight. No significant associations were observed between SES indicators and being overweight; however, prevalence of being overweight was higher among children whose mothers had less than a high school education (26.3%) than those whose mothers had higher education (11.0%–12.1%).

**Table 2.** Associations of SES indicators and maternal weight status with overweight in children aged 6 to 11 years (*n* = 397)

	Prevalence %	Crude OR (95% CI)	Adjusted^a^ OR (95% CI)
Maternal weight status			
Underweight	4.8	0.39 (0.09, 1.72)	—
Normal weight	11.5	1.00	—
Obese	25.9	2.72 (1.30, 5.71)^b^	—
Household income			
High	11.9	1.00	1.00
Middle	14.2	1.23 (0.59, 2.55)	1.21 (0.56, 2.59)
Low	4.4	0.34 (0.04, 2.82)	0.37 (0.04, 3.20)
Household expenditure			
High	12.0	1.00	1.00
Middle	10.1	0.84 (0.37, 1.91)	0.87 (0.37, 2.04)
Low	14.1	1.23 (0.57, 2.66)	1.45 (0.64, 3.26)
Maternal education			
>High school	12.1	1.00	1.00
High school	11.0	0.90 (0.44, 1.82)	0.85 (0.41, 1.78)
<High school	26.3	2.54 (0.77, 8.32)	2.20 (0.62, 7.81)
Paternal education			
>High school	10.8	1.00	1.00
High school	14.5	1.41 (0.71, 2.79)	1.24 (0.60, 2.57)
<High school	13.6	1.29 (0.33, 5.10)	0.98 (0.22, 4.28)
Maternal occupation			
Professional/manager	10.0	1.00	1.00
Sales/service/clerical	15.0	1.58 (0.57, 4.35)	1.69 (0.59, 4.90)
Security/transportation/labor	5.9	0.55 (0.06, 5.11)	0.51 (0.05, 5.01)
Others	13.8	1.41 (0.53, 3.77)	1.28 (0.46, 3.61)
Paternal occupation			
Professional/manager	10.8	1.00	1.00
Sales/service/clerical	11.0	1.00 (0.39, 2.55)	0.91 (0.34, 2.45)
Security/transportation/labor	16.2	1.60 (0.72, 3.58)	1.66 (0.72, 3.84)
Others	14.3	1.40 (0.27, 7.40)	1.03 (0.18, 6.02)

BMI, body mass index; CI, confidence interval; OR, odds ratio; SES, socioeconomic status.

^a^Adjusted for age, sex, and maternal BMI.

^b^*P* < 0.05.

Table 3 shows the ORs and 95% CIs of being overweight for adolescents. Similarly, adolescents with an obese mother were more likely to be overweight (OR 3.47; 95% CI, 1.49–8.07) than those whose mothers were normal weight. In contrast to the data for overweight children, the indicators of household economy (ie, household income and expenditure) were significantly associated with being overweight among adolescents. Adolescents living in middle-income households were more likely to be overweight (OR 2.73; 95% CI, 1.14–6.54) than those living in high-income households. The ORs remained significant after adjustment for confounders (OR 2.26; 95% CI, 1.01–5.67). Further, adolescents living in households with low expenditure levels were more likely to be overweight (OR 3.37; 95% CI, 1.25–9.06) than those living in households with high expenditure levels. Additionally, ORs remained significant after adjustment for confounders (OR 3.40; 95% CI, 1.20–9.60). We performed a sensitivity analysis using a different method for handling missing data and obtained similar results (eTable 1).

**Table 3.** Associations of SES indicators and maternal weight status with overweight in adolescents aged 12 to 18 years (*n* = 397)

	Prevalence %	Crude OR (95% CI)	Adjusted^a^ OR (95% CI)
Maternal weight status			
Underweight	1.8	0.23 (0.03, 1.81)	—
Normal weight	7.8	1.00	—
Obese	21.7	3.47 (1.49, 8.07)^b^	—
Household income			
High	5.6	1.00	1.00
Middle	14.1	2.73 (1.14, 6.54)^b^	2.26 (1.01, 5.67)^b^
Low	8.7	1.57 (0.29, 8.41)	1.49 (0.24, 9.16)
Household expenditure			
High	4.8	1.00	1.00
Middle	7.0	1.47 (0.49, 4.38)	1.46 (0.47, 4.61)
Low	15.1	3.37 (1.25, 9.06)^b^	3.40 (1.20, 9.60)^b^
Maternal education			
>High school	7.0	1.00	1.00
High school	9.3	1.37 (0.61, 3.08)	1.52 (0.65, 3.55)
<High school	18.2	2.92 (0.50, 17.04)	2.35 (0.37, 15.06)
Paternal education			
>High school	10.4	1.00	1.00
High school	7.6	0.73 (0.30, 1.74)	0.63 (0.25, 1.58)
<High school	16.7	1.74 (0.48, 6.34)	1.69 (0.42, 6.80)
Maternal occupation			
Professional/manager	8.3	1.00	1.00
Sales/service/clerical	9.8	1.24 (0.44, 3.50)	1.47 (0.48, 4.46)
Security/transportation/labor	16.7	2.14 (0.54, 8.53)	2.41 (0.56, 10.42)
Others	8.8	1.10 (0.34, 3.51)	0.99 (0.28, 3.49)
Paternal occupation			
Professional/manager	6.3	1.00	1.00
Sales/service/clerical	13.4	2.41 (0.79, 7.29)	1.93 (0.60, 6.21)
Security/transportation/labor	10.9	1.92 (0.66, 5.61)	1.64 (0.53, 5.03)
Others	10.7	1.94 (0.43, 8.74)	1.37 (0.28, 6.71)

BMI, body mass index; CI, confidence interval; OR, odds ratio; SES, socioeconomic status.

^a^Adjusted for age, sex, and maternal BMI.

^b^*P* < 0.05.

## DISCUSSION

Using nationally representative data, this study demonstrated that lower household income and expenditure were associated with risk of being overweight in Japanese adolescents. However, no significant associations were found between SES indicators and being overweight for Japanese children.

Consistent with reports of systematic reviews,^9^^,^^10^ our study found an inverse association between SES and being overweight among adolescents. However, a systematic review of cross-sectional studies in Western developed countries reported that such inverse associations were seen more often in children than in adolescents.^9^ The researchers also reported that parental education was more consistently inversely associated with being overweight than were parental occupation and income. Another systematic review of studies conducted in the United Kingdom reported that head-of-household occupation and maternal education were reliable determinants of childhood obesity.^10^ In contrast, the inverse association observed in the present study was seen only in adolescents, and low household economic status was associated with being overweight, rather than parental education and occupation.

What are the potential factors contributing to these differences observed between Japanese and Western societies? First, the health of adolescents may be more sensitive to differences in SES than that of children within the Japanese context, although further examination is needed to support this assumption. For example, because a larger proportion of public elementary schools than middle or high schools provide lunch for their students in Japan,^27^ school lunch may reduce the differences in overweight prevalence across SES groups among children. Second, a possible reason why a significant association between parental education and being overweight was not detected in our study was the study’s relatively small sample size. Indeed, our results revealed a higher prevalence of being overweight among both children and adolescents whose mothers had less than a high school education compared with those whose mothers had higher education. Third, the distribution and relative value of education attainment may differ according to the generation to which the parents belong,^28^ although some Japanese studies have suggested that educational attainment has had more effects on class identification in Japan since the 1990s.^29^ These differences may have underestimated the association between parental education and being overweight. Finally, occupational class may not be a reliable indicator of SES in Japan, unlike in Western countries.^30^ A previous review of SES and health in the Japanese population reported that occupational gradients in health were smaller in Japan than in other countries and that the magnitude and pattern of these gradients were inconsistent.^14^

In our analyses, we included participants with missing data on parental weight status and SES indicators because such a strategy would minimize biases caused by missing data in the results (estimates for missing data were not shown in the results). Participants with lower SES might have more missing data than those with higher SES. Thus, we did not use a list-wise deletion strategy because it would underestimate the association between SES and being overweight. We also could not use a multiple imputation strategy, as data were not missing at random. However, the missing indicator method used in this study is not a perfect tool to handle missing data and still gives biased estimates. The direction and size of the bias depended on the reason or mechanism of missingness.^31^

The mechanisms underlying the association between SES and being overweight among children and adolescents have yet to be established. However, the theoretical framework proposed by Sobal^32^ suggests that SES indicators may be related to obesity through dietary and exercise behaviors that contribute to a positive energy balance. This framework also proposes that each SES indicator may be related to obesity through different pathways: education is related to obesity through knowledge and beliefs; occupation, through lifestyle and shared values of appropriate body shapes; and income, through access to resources. Previous studies have indicated that individuals with higher incomes possess greater economic capacity to purchase healthier foods, such as fresh fruits and vegetables.^33^ Therefore, further research is needed to examine more thoroughly how access to food mediates observed correlations between household economy and overweight.

To our knowledge, this was the first study in Japan to demonstrate an association between SES and being overweight among children and adolescents, which was accomplished using individual-level data from two nationally representative surveys. However, our study had some limitations. First, due to the study’s cross-sectional nature, we were not able to determine the temporality of the association, although the possibility of reverse causality—that is, child’s overweight leads to low parental SES—was very unlikely. Second, participation rates were not very high in the NHNS, although the study samples were randomly selected. A previous study suggested that the low participation rate of younger adults in NHNS may lead to the loss of representativeness.^34^ Because participation rates for physical assessments were especially low, we compared the characteristics between participants with and without missing data on height and weight. As a result, we found that those with missing data on parental weight status and those whose fathers had less than a high school education were more likely to have missing data on personal height and weight than those without missing data (data not shown). This indicated selection bias in this study. Third, we could not consider a more detailed distribution of household income because it was assessed using the four categories in the NHNS. We were also not able to sufficiently examine the effects of low household income on the prevalence of overweight among Japanese children and adolescents due to the category’s small cell size. Fourth, unmeasured factors may have affected the associations we observed. Commonly identified examples of such confounders include psychological problems.^35^ However, if psychological problems mediate the association between SES and being overweight, there is the risk of over-adjusting for them in analyses. Finally, the study’s estimates of ORs for being overweight need to be interpreted with some degree of caution because of the potential for high variability associated with the small sample size.

In conclusion, the results of this population-based cross-sectional study indicated that parental SES was associated with overweight in adolescents. Prospective studies are required to establish a causal link between SES and overweight. Additionally, SES should be considered in the prevention of childhood and adolescent obesity.

## ONLINE ONLY MATERIAL

eTable 1. Associations of SES indicators and maternal weight status with overweight: model-wise deletion analysis.
