# Supplementary material for: Socioeconomic Status and Overweight: A Population-Based Cross-Sectional Study of Japanese Children and Adolescents
Source: J Epidemiol. 2015 Jul 5;25(7):463–9. doi: 10.2188/jea.JE20140108 (PMC4483371; doi:10.2188/jea.JE20140108)
Supplement: eTable 1. [file je-25-463-s001.pdf]

eTable 1. Associations of SES indicators and maternal weight status with overweight: model-wise deletion analysis

	Aged 6 to 11 years Adjusted OR (95% CI)*		Aged 12 to 18 years Adjusted OR (95% CI)*	
Household income	(n = 328)		(n = 314)	
High	1.00		1.00	
Middle	1.14	(0.53, 2.46)	3.14	(1.15, 8.61)†
Low	0.38	(0.04, 3.30)	1.09	(0.10, 11.54)
Household expenditure	(n = 354)		(n = 341)	
High	1.00		1.00	
Middle	0.87	(0.37, 2.05)	1.21	(0.37, 3.97)
Low	1.39	(0.61, 3.16)	3.29	(1.13, 9.56)†
Maternal Education	(n = 340)		(n = 329)	
>High school	1.00		1.00	
High school	0.86	(0.41, 1.81)	1.33	(0.56, 3.17)
<High school	1.52	(0.39, 5.96)	2.51	(0.39, 16.12)
Paternal Education	(n = 324)		(n = 284)	
>High school	1.00		1.00	
High school	1.19	(0.57, 2.50)	0.63	(0.25, 1.61)
<High school	1.03	(0.23, 4.62)	1.63	(0.36, 7.38)
Maternal Occupation	(n = 363)		(n = 352)	
Professional/manager	1.00		1.00	
Sales/service/clerical	1.71	(0.59, 4.94)	1.20	(0.39, 3.69)
Security/transportation/labour	0.51	(0.05, 5.00)	2.07	(0.48, 8.86)
Others	1.29	(0.46, 3.60)	0.86	(0.25, 3.00)
Paternal Occupation	(n = 315)		(n = 278)	
Professional/manager	1.00		1.00	
Sales/service/clerical	0.90	(0.33, 2.45)	1.71	(0.51, 5.76)
Security/transportation/labour	1.59	(0.67, 3.75)	1.35	(0.41, 4.44)
Others	1.06	(0.18, 6.33)	1.52	(0.30, 7.66)

BMI, body mass index; CI, confidence interval; OR, odds ratio; SES, socioeconomic status.

*Adjusted for age, sex, and maternal BMI

†$p < 0.05$
